# Report on the Sanitary Condition of the City, Etc.

**Published:** 1867-06

**Authors:** N. S. Davis

**Affiliations:** Member of Sanitary Committee


ARTICLE XXV.

### REPORT ON THE SANITARY CONDITION OF THE CITY, AND THE PREVALENCE OF DISEASES, FROM NOV. 1st, 1866, TO APRIL 1st, 1867.

By N. S. DAVIS, M.D., Member of Sanitary Committee.

Read to the Chicago Medical Society, April 27th, 1867.

In my last report an account of the sanitary condition of the city was given up to the 24th of October, 1866.

Up to that date the epidemic, cholera, had continued to prevail moderately; but on the 20th of that month the atmosphere had become cool, cloudy, with some rain. On the 21st there fell copious showers, with lightning, and a severe gale of wind from the west.

The 22d was cool, nearly clear, with a sharp frost at night; and on the 23d sufficient snow fell to whiten the surface of the ground. From this date the prevalence of cholera declined so rapidly, that after the first of November hardly a single new case was to be found in the city. The month of November was cool, for the most part pleasant, and presented no atmospheric or meteorological peculiarities worthy of note. The prevalence of sickness, and the rate of mortality, were but very little above that of the most healthy seasons, while in October the gross mortality was 1170, of which 673 was from cholera, and 115 from other affections of the bowets; in November the gross mortality was only 382, of which 12 were attributed to cholera, and only 28 to other bowel-affections. The total mortality for the same months in 1865 were: for October 360, and November 299.

During the months of December, January, February, and March, there was an unusual predominance of steady, moderate, cold, and dry weather, with unusually frequent and abundant falls of snow. And there was a correspondingly low ratio of sickness and mortality throughout all these months, as shown by the following figures:—Total mortality for December, 309; January, 299; February, 255; March, 280.

These rates are below the average mortality of the same months for a series of years. From the subsidence of cholera during the last week of October to the first of April, 1867, no disease of an epidemic character has prevailed in our city. We will, therefore, close our labor as a member of your committee on the sanitary condition of the city for the year ending April 1st, 1867, with a complete statement of the monthly mortality for the year 1866:

| | |
|---|---|
| January, | 293 |
| February, | 260 |
| March, | 254 |
| April, | 278 |
| May, | 275 |
| June, | 319 |
| July, | 706 |
| August, | 940 |
| September, | 739 |
| October, | 1170 |
| November, | 382 |
| December, | 309 |
| Total, | 5925 |

Excess of mortality over 1865, 2262, of which 990 were cholera.

